# Characteristics of the ErmK Protein of Bacillus halodurans C-125

**DOI:** 10.1128/spectrum.02598-22

**Published:** 2022-12-13

**Authors:** Sung Keun Kim, Yu Hong Min, Hyung Jong Jin

**Affiliations:** a Department of Bioscience and Biotechnology, The University of Suwon, Hwaseong City, South Korea; b College of Health and Welfare, Daegu Haany University, Gyeongsangbuk-Do, South Korea; University of Guelph

**Keywords:** extromophile, antibiotic resistance, fitness cost, methylases

## Abstract

Bacillus halodurans C-125 is an alkaliphilic microorganism that grows best at pH 10 to 10.5. *B*. *halodurans* C-125 harbors the *erm* (erythromycin resistance methylase) gene as well as the *mphB* (macrolide phosphotransferase) and putative *mef* (macrolide efflux) genes, which confer resistance to macrolide, lincosamide, and streptogramin B (MLS_B_) antibiotics. The Erm protein expressed in *B*. *halodurans* C-125 could be classified as ErmK because it shares 66.2% and 61.2% amino acid sequence identity with the closest ErmD and Erm(34), respectively. ErmK can be regarded as a dimethylase, as evidenced by reverse transcriptase analysis and the antibiotic resistance profile exhibited by E. coli expressing *ermK*. Although ErmK showed one-third or less *in vitro* methylating activity compared to ErmC′, E. coli cells expressing ErmK exhibited comparable resistance to erythromycin and tylosin, and a similar dimethylation proportion of 23S rRNA due to the higher expression rate in a T7 promoter-mediated expression system. The less efficient methylation activity of ErmK might reflect an adaption to mitigate the fitness cost caused by dimethylation through the Erm protein presumably because *B*. *halodurans* C-125 has less probability to encounter the antibiotics in its favorable growth conditions and grows retardedly in neutral environments.

**IMPORTANCE** Erm proteins confer MLS_B_ antibiotic resistance (minimal inhibitory concentration [MIC] value up to 4,096 μg/mL) on microorganisms ranging from antibiotic producers to pathogens, imposing one of the most pressing threats to clinics. Therefore, Erm proteins have long been speculated to be plausible targets for developing inhibitor(s). In our laboratory, it has been noticed that there are variations in enzymatic activity among the Erm proteins, Erm in antibiotic producers being better than that in pathogens. In this study, it has been observed that Erm protein in *B*. *halodurans* C-125 extremophile is a novel member of Erm protein and acts more laggardly, compared to that in pathogen. While this sluggishness of Erm protein in extremophile might be evolved to reduce the fitness cost incurred by Erm activity adapting to its environments, this feature could be exploited to develop the more potent and/or efficacious drug to combat formidably problematic antibiotic-resistant pathogens.

## INTRODUCTION

Although some microorganisms grow well at more than one pH depending on the growth conditions, generally, microbes classified as alkaliphiles grow very well or optimally at pHs above 9, and often grow well at pHs between 10 and 12. Alkaliphiles cannot grow or grow only slowly at the near neutral pH value of 6.5. Despite these properties, alkaliphilic microorganisms coexist with neutrophilic microorganisms as well as occupying specific extreme environments in nature. The abundance of alkaliphiles in ordinary neutral soil is one-tenth to one-hundredth of the population of neutrophilic microorganisms (1). The alkaliphilic Bacillus halodurans C-125 was isolated from soil, and its optimum pH for growth is pH 10 to 10.5. Although it grows at even 55°C, the optimum temperature for this microorganism is about 37°C at pH 10.0 (2). It grows very slowly below pH 7.6, but the addition of 0.2 M NaCl caused dramatic growth even at pH 7.2 (3). Furthermore, this microorganism is actively motile when grown at an alkaline pH and has been found to grow in the presence of high concentrations of sodium chloride (up to 12%) (4). Although *B. halodurans* C-125 could grow in environments with relatively high salinity, it could not be classified as haloalkaliphiles because it can also grow in even higher salinities (up to 33% [wt/vol] NaCl) to be called haloalkaliphile. However, *B. halodurans* C-125 can be one of the extremophilic bacteria since pH for its optimal growth is around 10. *B. halodurans* C-125 shares some similarities with Bacillus subtilis in terms of size of genome, G+C content of genomic DNA, and the physiological properties adopted for taxonomical identification, except for the alkaline phenotype. But recently, *B. halodurans* has been reclassified as *Alkalihalobacillus halodurans* (5, 6). The whole genome of *B*. *halodurans* C-125 was sequenced in the year 2000 second to B. subtilis in the genus *Bacillus* (7). Among the functionally assigned 2,141 protein-coding sequences (CDSs) out of the 4,066 predicted CDSs, a CDS for rRNA adenine N-6-methyltransferase (named BH0380) has been identified, and presumably constitutes the operon with the CDS for macrolide 2’-phosphotransferase (*mphB*). In addition to these CDSs, a CDS for a macrolide efflux transporter was also identified in the different location of the genome (named BH2749). Therefore, *B*. *halodurans* C-125 contains three different resistance CDSs related to macrolide antibiotics. For the first time, the product of BH0380 was recognized and adopted into the phylogenetic analysis of Erm proteins under the name Erm_BACHA in our laboratory without any experimental confirmation of its function (8). When part of *B*. *halodurans* C-125 chromosome had been sequenced, and the gene and product of BH0380 which named after the completion of the sequencing of whole chromosome-had been named *ermK* and ErmK (9), respectively, based on the sequence homology with other Erm protein (10) (see below for detail). After the completion of sequencing of the whole chromosome, the product of BH0380 was designated ErmK in CARD (comprehensive antibiotic resistance database (11) and the following report (12). To the best of our knowledge, the function of BH0380 has not been elucidated and/or confirmed clearly by experimental procedures.

Erm proteins are classified into monomethyltransferase (such as ErmN, previously named TlrD [13]) and dimethyltransferases (such as ErmC′ [14]), and act on the exocyclic amino group of a specific adenine residue (A2058, Escherichia coli coordinate) to reduce the affinity of MLS_B_ antibiotics, thereby conferring resistance on microorganisms harboring Erm proteins (8, 15). While monomethyltransferases confer high-level resistance to lincosamides and medium or low level resistance to macrolide and streptogramin B antibiotics, microorganisms expressing dimethyltransferases exhibit high level resistance to all three groups of antibiotics (16–18). However, it is not possible to differentiate between a monomethyltransferase and a dimethyltransferase based on sequence comparison (17, 19) or using a phylogenetic tree construction algorithm (8). The clarification of the methylation capability of each Erm protein depends on experimental demonstration. Therefore, the exact functional role of BH0380 should be elucidated via experimental procedures and if possible, its function in extremophile could be uncovered along with this. In this regard, characterization of the product of BH0380 from *B*. *halodurans* C-125, extremophile was carried out in this study.

## RESULTS

### Relatedness of ErmK protein and its gene to other Erm proteins and *erm* genes.

**FIG 1:**
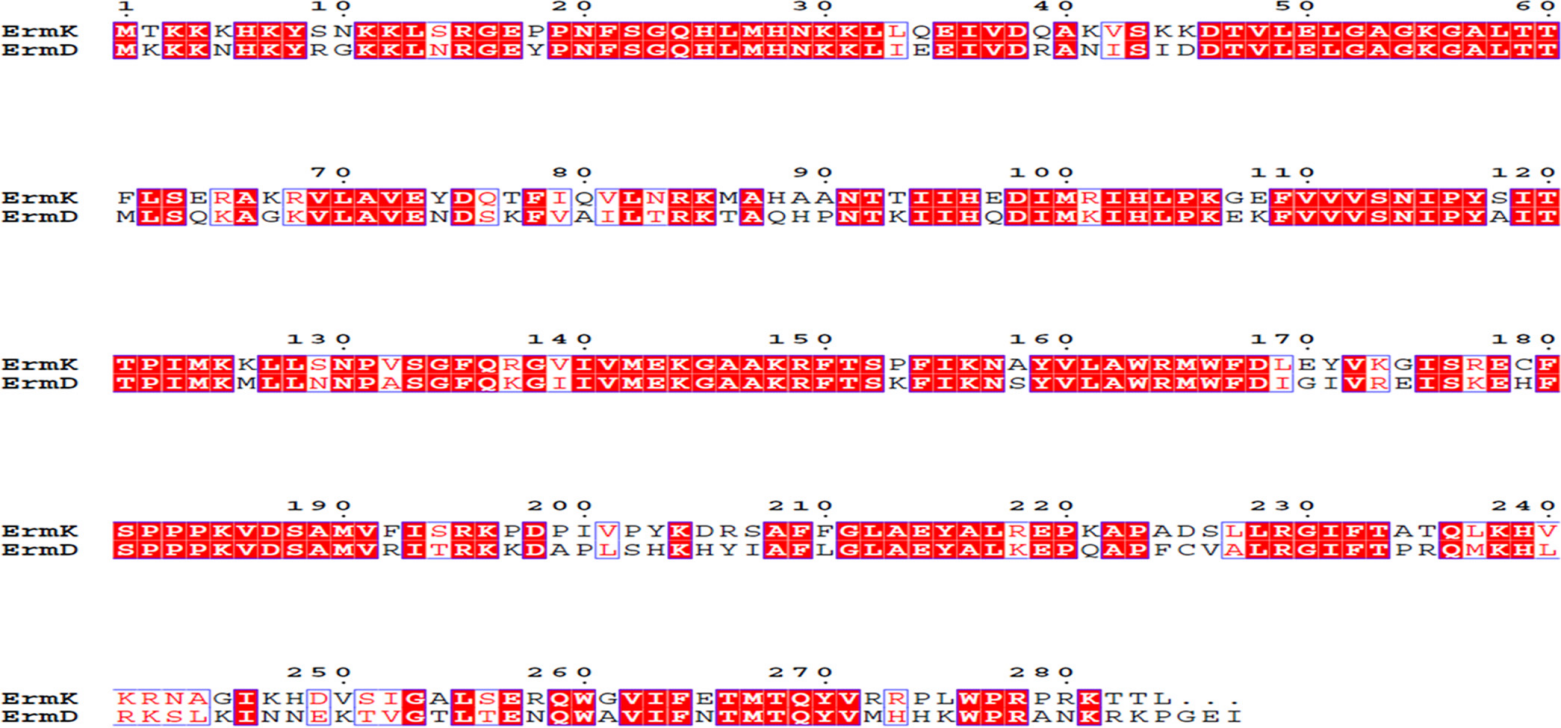
Sequence alignment of ErmK and ErmD. ErmK is originated from *B*. *halodurans* C-125, and ErmD is from B. licheniformis (42) and one of the closest members to ErmK, the other being Erm(34). When the sequences of two proteins were aligned, 188 positions were recognized to be occupied with the same amino acids so that two proteins exhibited 66.2% amino acid sequence identity which is less than 80%. Therefore, along with the sequence comparison result of Erm(34) and ErmK, 61.2% amino acid sequence identity (Fig. S1), it could be considered to be reasonable and correct that ErmK might be classified as a new member of Erm protein and given a new letter designation of ErmK. The sequence alignment was generated using CLUSTALX2 (40), and the resulting alignment was visualized using ESPript (41).

As described in the introduction, when part of the *B*. *halodurans* C-125 chromosome was sequenced, a new class of *erm* gene and its product was inferred through homology and were named *ermK* and ErmK (accession number Q9RC37) after sequence homology comparison with similar other Erm protein, presumably Erm(D) from B. licheniformis or B. anthracis (not B. subtilis because the length of its amino acid sequence was 287). After completion of whole-genome sequencing, the gene was named BH0380. For the first time, ErmK was phylogenetically analyzed in our lab under the name Erm_BACHA and formed one branch with Erm(D) and Erm(34) on each side(8). The same observation was obtained in a recently reported study (12). To verify Erm_BACHA’s new class designation, its amno acid sequence identity with Erm(D) and Erm(34) should be compared. Compared, Erm_BACHA exhibited 66.2% amino acid sequence identity with ErmD from B. licheniformis (Fig. 1) and 61.2% with Erm(34) from *B*. *clausii* (Fig. S1). Assigning the product of BH0380 to a new class and giving a new letter designation (*ermK* and ErmK) appeared to be reasonable and correct.

### Expression of Erm proteins in E. coli.

**FIG 2:**
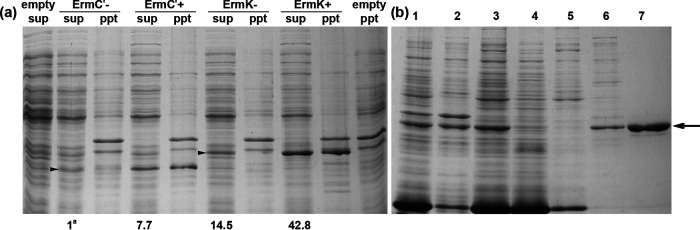
SDS-PAGE analysis of Erm protein expression (ErmC′ and ErmK) in E. coli and purification (ErmK). (a) Proteins were expressed in the presence of ITPG, inducer (+) or without it (−). To separate the soluble proteins (sup) from the precipitated proteins (ppt, inclusion body fraction), centrifugation (20,000 × *g*, 30 min) was carried out after disruption of cells by freezing and thawing. Arrowhead indicates ErmC′ (28.907 kDa, calculated) and ErmK (32.336 kDa), respectively. a, the relative amount of each protein was measured using Multi Gauge (ver3.0). The protein amount of ErmC′ expressed in soluble fraction without inducer was set to 1 and the amount of other protein was indicated in the relative amount to it. (b) The soluble fraction of protein (ErmK) was purified on immobilized Ni^2+^affinity column. Lane 1, total cell proteins; lane 2, inclusion body fraction; lane 3, supernatant fraction of lysate; lane 4, affinity runthrough; lane 5, 1 × binding buffer column wash; lane 6, 100 mM imidazole column wash; lane 7, purified soluble ErmK protein (arrowed).

ErmC′ and ErmK were successfully expressed in E. coli BL21(DE3) with and without IPTG induction (Fig. 2a). When expressed without IPTG induction (leaky expression), most of the expressed proteins resided in the soluble protein fraction with no or minute amounts detected in inclusion body fraction after SDS-PAGE. With IPTG induction, both the soluble protein and precipitated one increased dramatically. Soluble, overexpressed ErmK constituted around 12% of the total E. coli soluble proteins and the ratio of soluble to insoluble inclusion fraction of overexpressed protein was around 6 to 4. Thirty-five milligrams of purified His6-tagged ErmK could be obtained from 1 L of induced culture (Fig. 2b). When the protein level of ErmC′ expressed in soluble form without inducer was set to 1, the relative amount of soluble ErmC′ with induction was 7.7. While the relative amount of ErmK was 14.5 without induction, that of ErmK with induction was 42.8. Both ErmC′ and ErmK could be overexpressed in sufficient quantity to purify and evaluate its methylation activity *in vitro*. ErmN was successfully expressed in E. coli BL21(DE3) in a previous study (20), and it could be expressed with and without IPTG induction in soluble form (data not shown).

### Activity of Erm proteins in E. coli.

**FIG 3:**
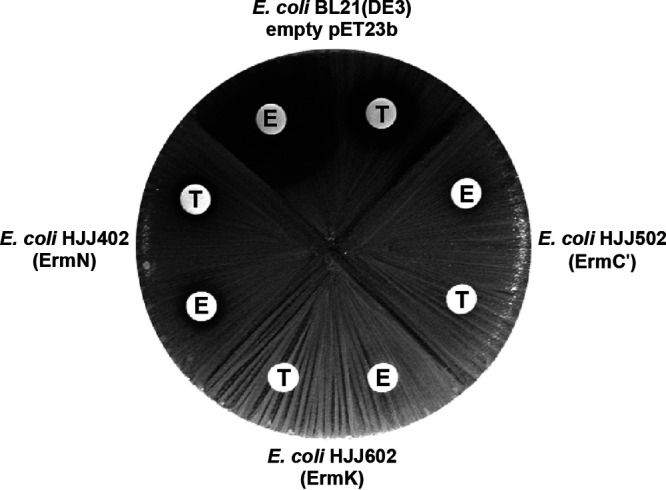
Antibiotic susceptibility assay with E. coli cells expressing ErmC′, ErmN and ErmK and E. coli containing only empty vector. Overnight grown cells was spread on quartered agar plate and circular filter paper containing 1,000 μg of tylosin and erythromycin was placed in the middle of culture. E. coli cells with empty vector exhibit reasonably wide inhibition zone caused by each antibiotic. Presumably tylosin experiences more outer membrane permeability barrier than erythromycin, forming smaller inhibition zone. With monomethylation (ErmN), the inhibition around each antibiotic reduced to form smaller but similar size of inhibition zone. With ErmC′ and ErmK, no inhibition zone could be observed indicating ErmK could be dimethyltransferase.

Unlike drug-permeable E. coli AS19, E. coli BL21(DE3) is not a strain that macrolide antibiotics can easily penetrate, because of the penetration barrier of the outer membrane (21). Erythromycin seemed to penetrate the E. coli outer membrane more easily than tylosin: when the circular filter paper containing the same amount of antibiotic (1,000 μg) was applied to the center of the culture, a much larger inhibition zone could be observed with erythromycin in E. coli lawn harboring empty vector (Fig. 3). However, once ErmN was expressed in the cell, the inhibition zone was significantly reduced and only a small residual clear zone remained with both erythromycin and tylosin. When ErmC′ and ErmK were expressed, no inhibition zone could be observed even in the presence of erythromycin and tylosin (Fig. 3).

### Degree of methylation by ErmK.

**FIG 4:**
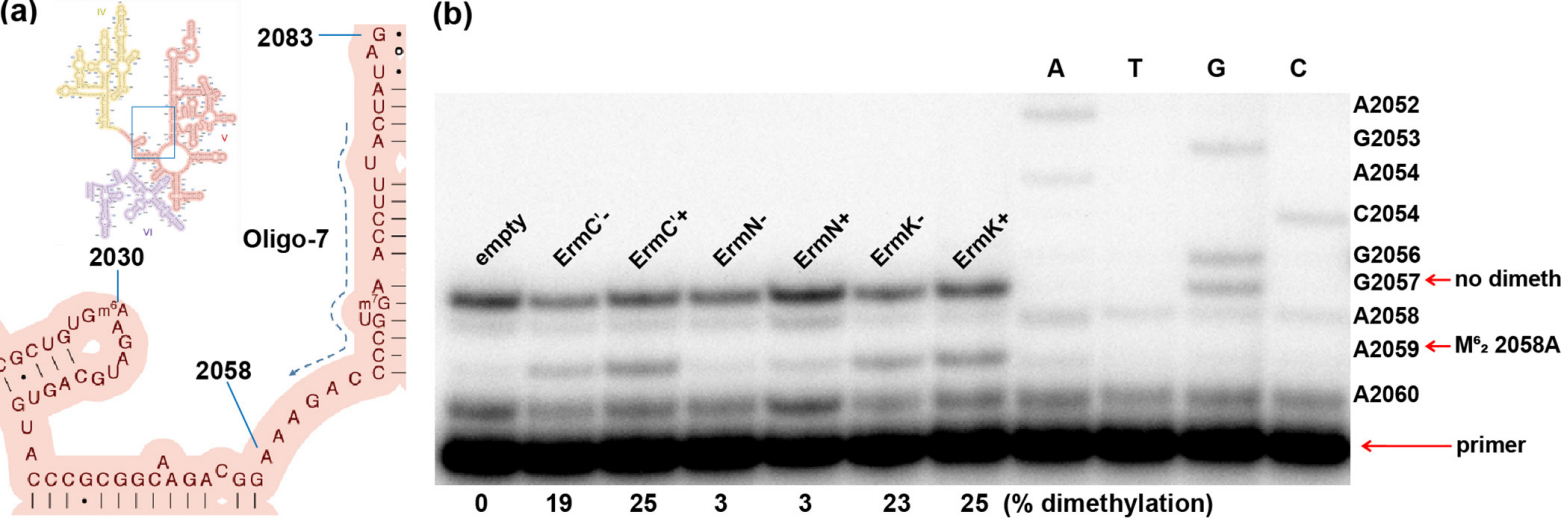
Autoradiogram of reverse transcripts of *in vivo*-methylated 23S rRNA in the presence of various Erm proteins. The rRNA was isolated from E. coli cells with or without IPTG induction which harbor plasmid empty pET23b, pHJJ402 (ErmN), pHJJ502 (ErmC′) and pHJJ602 (ErmK). All the 23S rRNAs were analyzed in the same manner by extension with reverse transcriptase from a primer (oligo-7) complementary to nucleotides 2061 to 2078 in E. coli 23S rRNA to assess the degree of methylation. Primer extension should start at A2060 and was designed to terminate at G2057 by the addition of ddCTP if there was no methylation (empty vector) or monomethylation (ErmN) at A2058 (no dimeth). The reverse transcription proceeded in the presence of only dTTP and ddCTP. But dimethyation at A2058 induced primer extension termination at A2059 (M^6^_2_2058A). The relative amounts of dimethylated and unmethylated or monomethylated RNA was inferred from the ratio between band intensity at A2059 and band intensity at A2057 plus that at A2059 and shown at the bottom of the autoradiogram (% dimethylation). Analysis with induced strain is indicated by + and that of leaky expression (without induction) is indicated by −. The 23S rRNA template for the dideoxy-sequencing reactions (ATGC) was from a sensitive E. coli strain harboring plasmid pET23b.

According to the antibiotic resistance profiles of E. coli strains expressing ErmK, ErmN, and ErmC′ and E. coli harboring the empty vector (Fig. 3), ErmK carried out the dimethylation reaction at a specific residue (A2058, E. coli coordinate). To confirm this observation, the degree of methylation at A2058 was assessed by reverse transcription. While reverse transcription on either an unmethylated or monomethylated rRNA template is not affected and proceeds until it is terminated by incorporation of ddCTP at G2057 (the first G residue which reverse transcriptase encounters during transcription), dimethylation at A2058 terminates the progress of transcription at position 2059 producing a gel band that can be quantified. As can be observed in Fig. 4b, compared to the reverse transcription reaction using rRNA from the strain carrying the empty vector and ErmN known as a monomethylase, a clear band originated from reverse transcription arrest (A2059) before the dimethylated A2058 could be observed with ErmK and ErmC′, corroborating the observation obtained from the antibiotic resistance profile test. To measure what portion of rRNA might be dimethylated by expressed ErmK and ErmC′ in E. coli, the ratio between the band intensity at A2059 and the band intensity at A2057 plus that at A2059 was determined because all the extended should be arrested at either A2059 or A2057. In leaky expressed cells, ErmK dimethylated 23% of rRNA while ErmC′ dimethylated 19% of rRNA. However, in IPTG-induced cells, similar dimethylation rates could be observed (25%).

### Comparison of ErmK activity to that of ErmC′ *in vitro*.

**FIG 5:**
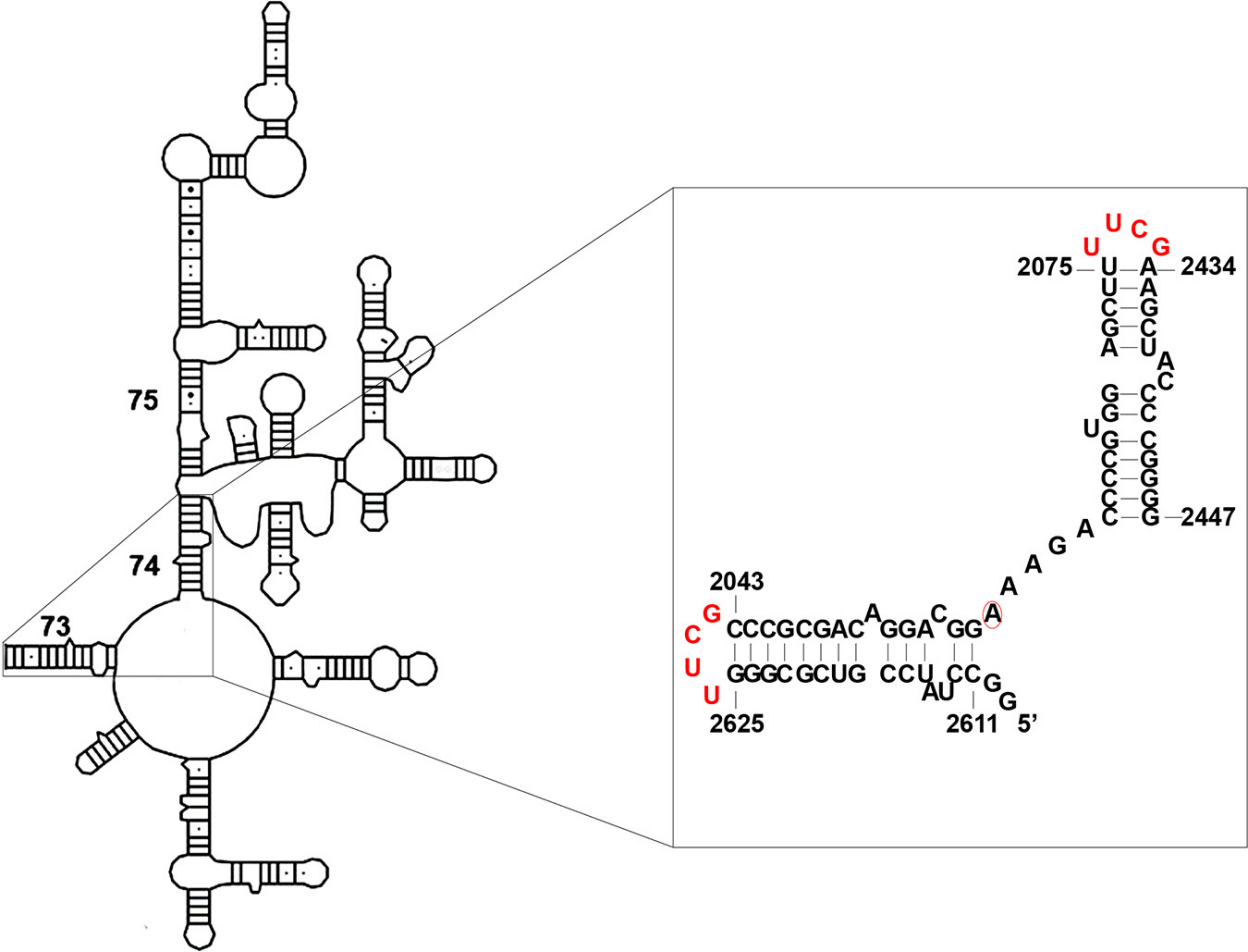
72 nt substrate rRNA sequence used for measuring the methyl group transferring activity of ErmK and ErmC′. 72 nt substrate RNA is composed of helix 73 and 74 and from B. subtilis BD170. To facilitate the production of substrate RNA, the ends of helix 73 and 74 were capped with UUCG tetraloop (red) that all the sequences reside on one string of RNA strand. The methylatable adenine is encircled in red.

Domain V has been thought to contain all the structural components to be recognized and methylated by Erm proteins which is a complete substrate for Erm proteins (22, 23). 72 nt substrate RNA is composed of helices 73 and 74 and can be obtained as one strand of RNA by *in vitro* transcription because the ends of helix 73 and 74 were capped with a UUCG tetraloop (Fig. 5). When 72 nt RNA substrate was used, it exhibited around 60% substrate activity compared to domain V through ErmS (H. J. Lee and H. J. Jin, unpublished result). ErmK and ErmC′ produced 1,818 cpm and 5,365 cpm by transferring the tritium-labeled methyl group from *S*-adenosyl-l-methionine (SAM) to substrate RNA (72 nt) under standard assay conditions, respectively.

## DISCUSSION

The outer membrane of Gram-negative bacteria, including Enterobacteriaceae (e.g., E. coli) acts as a relatively effective permeability barrier against all hydrophobic antibiotics, including erythromycin (21, 24). Although this principle can be applied to E. coli BL21(DE3), it seems to be possible to differentiate the macrolide resistance caused by monomethylation, dimethylation and non-methylation in the presence of a large amount of antibiotics (1,000 μg) by employing E. coli strain equipped with a permeability barrier. When a circular filter paper containing the same amount of tylosin or erythromycin (1,000 μg) was placed in the middle of culture of E. coli harboring the empty vector, a reasonably sized clear zone, formed by the inhibitory activity of antibiotics could be observed. However, a much larger inhibition zone was exhibited with erythromycin compared to that with tylosin, probably suggesting that tylosin was experiencing more outer membrane permeability barrier. Once expressed in E. coli BL21(DE3), ErmN reduced both inhibition zones formed with tylosin and erythromycin and obtained similar-sized inhibition zones (Fig. 3). Tylosin and erythromycin exhibit different levels of resistance to monomethylation at A2058: while monomethylation could confer medium resistance to erythromycin, it causes low resistance to tylosin. However, when monomethylation at A2058 is combined with the monomethylation at G748 within the loop of 23S rRNA hairpin 35, the resistance to tylosin increases to a moderately high level of resistance (25). Like many other Gram-negative bacteria, E. coli BL21(DE3) may carry another functional methyl group transferring enzyme (RlmA^II^) at G745 (26) and sometimes it increases resistance to tylosin a little bit, together with monomethylation at A2058 even though methylation at G745 does not interfere with the binding of tylosin to the ribosome. However, this effect could not fully explain the observed phenomenon with tylosin treatment, while the reduction in the size of the inhibition zone with erythromycin treatment seemed reasonable. The probable explanation at the moment might be synergy between methylation(s) of certain nucleotide(s) and the outer membrane permeability barrier posed by Gram-negative bacteria because tylosin may experience a greater permeability barrier in the outer membrane of E. coli. Expression of ErmC′ or ErmK did not confer any inhibition zone around the disk containing 1,000 μg of tylosin and erythromycin, indicating that both Erm proteins are dimethylases because only dimethylation at A2058 can confer high resistance to both antibiotics. This observation is congruent with the result obtained from the determination of the degree of methylation by reverse transcription using rRNAs from E. coli cells expressing Erm proteins (Fig. 4). Monomethylation or nonmethylation at A2058 results in reverse transcription passing over A2058 and terminating at G2057 by incorporation of ddCTP, while dimethylation at A2058 arrests the progress of reverse transcription at A2059. Furthermore, the band intensity obtained from noninduced strains harboring *ermC′* and *ermK* was weaker than that obtained from IPTG-induced strains, indicating more proteins and more dimethylated rRNA at A2058 and supporting the dimethylating activity of ErmK. Although domain V (>600 nucleotides) of bacterial 23S rRNA has been considered to contain all the structural elements to be recognized and methylated by Erm proteins, a relatively large RNA transcript should be employed when domain V is used to test the activity of an Erm protein as a substrate. A simpler but efficient substrate (72 nt) could be designed to make the Erm protein activity assay easier. The 72 nt RNA substrate composed of helices 73 and 74 (in which the end of each helix was capped with the tetraloop, UUCG) exhibited 60% activity compared to that obtained with domain V with ErmS. In the previous report (27), ErmS was shown to recognize the shortest motif of the RNA substrate to methylate and exhibit the highest activity with the substrate equipped with helix 73 and its surrounding region that it might reflect the potential for substrate activity of each participating nucleotide more clearly. In this sense, 72 nt RNA might be good enough to play a distinguishing role in comparing Erm proteins for their methylation activity, despite its relatively small size. Two salts, KCl and MgCl_2_, were included in the methylation buffer. ErmC′ tended to be sensitive to the variation in salt concentration (with a large dependence of the methylation activity on MgCl_2_ and a relatively smaller dependence on KCl). The highest activity without MgCl_2_ was 29,240 cpm, 10 times higher activity than the activity of ErmK and the lowest activity was at 10 mM MgCl_2_ (one hundredth of the highest activity, 268 cpm, one quarter of the activity relative to ErmK). From no KCl (2,414 cpm) up to 40 mM KCl (5,365 cpm), the methylating activity increased gradually, but at 80 mM KCl, the activity dropped suddenly to one fifth of the activity at 40 mM KCl (1,020 cpm, a little more than half the activity relative to ErmK). In contrast, ErmK was relatively indifferent to the variations in salt concentration in the assay buffer. ErmK showed the highest activity without MgCl_2_ (2,982 cpm) as ErmC′ and, with increasing MgCl_2_ concentration, the methylating activity decreased accordingly, exhibiting around one third of the highest activity at 10 mM MgCl_2_ (1,164 cpm). Furthermore, for the variation of KCl from 0 to 80 mM, quite similar activity (around 1,818 cpm) was shown by ErmK. In summary, ErmC′ showed higher methylation activity compared to ErmK, except for the two extreme cases: 10 mM MgCl_2_ and 80 mM KCl. In the typical assay condition containing 4 mM MgCl_2_ and 40 mM KCl, ErmC′ and ErmK produced 5,365 cpm and 1,818 cpm, respectively, with ErmC′ generating around three times more methylated RNA than ErmK. Although ErmK exhibited one-third or less of the activity of ErmC′, it conferred resistance to large amount of erythromycin (filter paper containing 1,000 μg) on E. coli expressing it to a similar extent to E. coli expressing ErmC′ (Fig. 3), which could be observed without IPTG induction. Presumably, this is possible because in E. coli cells, ErmK is expressed 14.5 times more without IPTG induction (Fig. 2a). And this might also be reflected in the dimethylation proportion of A2058 in E. coli expressing ErmC′ (19%) and ErmK (23%) in the absence of IPTG (Fig. 4).

Dimethylation at A2058 by Erm proteins, which increases hydrophobicity and prevents the hydrogen bonding interaction of the exocyclic amino group, has been known to cause fitness cost by affecting the expression of a subset of cellular polypeptides. Since A2058 is located in a part of the nascent peptide exit tunnel, which is known to be involved in the recognition of the nascent peptide and in the modulation of translation, its dimethylation alters the ribosomal interaction with a defined nascent peptide sequence within the tunnel so that translation of a polypeptide harboring a certain peptide sequence at the appropriate site could be modified (28–30). Macrolide antibiotics are inactivated in highly acidic and highly basic environments (pH < 4 or >10). By definition, alkaliphilic microorganisms grow optimally or very well above pH 9, often between 10 and 12, but cannot grow or grow slowly at the near neutral pH value of 6.5. However, the existence frequency of alkaliphiles in ordinary, neutral soil samples is one tenth to hundredth of the population of neutrophils, indicating that alkaliphiles coexist with neutrophiles, as well as occupying extreme environments in nature (1). Although *B*. *halodurans* C-125 was isolated from soil, it is an alkaliphilic microorganism. Therefore, the low activity of ErmK compared to ErmC′ could be another measure developed by this extremophile to avoid the unnecessary fitness cost caused by the dimethylation of A2058, presumably due to the relatively low probability of encountering the antibiotics in the favorable growth conditions and retarded growth in neutral environment. Similar but more detailed observations have been reported previously (27): activity among Erm proteins is varied, that is Erm protein activity adapts to the needs posed by stimuli from the environment with distinct and specific features; for example, in the recognition of the minimal part of domain V for methylation and the decrease in substrate activity upon the same deletion from a certain substrate of RNA resulting in different methylation activity to a defined substrate RNA. Furthermore, in addition to its laggardly activity, the regulatory region of ErmK seems to exhibit less efficient inducibility of *ermK* compared to *ermC′* (S. K. Kim and H. J. Jin, unpublished results).

## MATERIALS AND METHODS

### Strains, plasmids, primers, and synthesized gene.

The bacterial strains and plasmids that were used in this study are listed in Table 1. Oligonucleotide primers and synthesized gene that were used are listed in Table 2.

**TABLE 1:** Bacterial strains and plasmids

Bacterial strain or plasmid	Description	Reference or source
Bacterial strains		
E. coli DH5α	Transformable host for plasmid construction	Novagen
E. coli BL21(DE3)	Host for plasmid expression vectors that utilize the T7 promoter; possess T7 RNA polymerase gene under *lac* control	Novagen
E. coli HJJ402	E. coli BL21(DE3) carrying plasmid pHJ402	20
E. coli HJJ502	E. coli BL21(DE3) carrying plasmid pHJJ502	This work
E. coli HJJ602	E. coli BL21(DE3) carrying plasmid pHJJ602	This work
B. subtilis BD1109	Strain carrying plasmid pIM13	33
B. halodurans C-125	Source of DNA template for obtaining *ermK* by PCR	7
Plasmids		
pHJJ402	pET23b containing *ermN Nde*I–*Xho*I gene cartridge	20
pHJJ502	pET23b containing *ermC′ Nde*I–*Xho*I gene cartridge	This work
pHJJ602	pET23b containing *ermK Nde*I–*Xho*I gene cartridge	This work
pET23b	Vector for high-level expression under T7 polymerase control; allows introduction of a His tag for protein purification by Ni^2+^ affinity chromatography	Novagen
pIM13	Source of DNA template for obtaining *ermC*′ by PCR	33

**TABLE 2:** Deoxyoligonucleotides and sequence of synthesized gene used in this study

Deoxyoligo-nucleotide	Sequence (5′ → 3′)	Description
Oligo-1	5′GGAATTCCATATGACCAAAAAAAAACACAAATACAGCAATAAAAAACTTAG3′	51-mer forward primer for *ermK* cloning, containing *Nde*I recognition site (underlined).
Oligo-2	5′*CGAGCACATA****A****GCATTTTTGA*TGAAAGGCGA3′	31-mer reverse primer for 5′ end part of *ermK* cloning, to change natural *Nde*I site by overlap extension PCR. Bold letter denotes mutagenized nucleotide and italicized letters overlapping sequence here and in oligo-3.
Oligo-3	5′*TCAAAAATGC****T****TATGTGCTCG*CATGGCGGATG3′	32-mer forward primer for 3′ end part of *ermK* cloning, to mutagenize intrinsic *Nde*I site. A mutagenized nucleotide and overlapping sequence is denoted by bold and italicized letter, respectively.
Oligo-4	5′GGCCTCGAGTAATGTTGTCTTTCTCGGTCG3′	30-mer reverse primer for *ermK* cloning, containing *Xho*I recognition site (underlined).
Oligo-5	5′GGAATTCCATATGAACGAAAAAAACATCAAACACTCTC3′	38-mer forward primer for *ermC′* cloning, containing *Nde*I recognition site (underlined).
Oligo-6	5′CCGCTCGAGCTTATTAAATAATTTATA3′	27-mer reverse primer for *ermC′* cloning, containing *Xho*I recognition site (underlined).
Oligo-7	5′GTAAAGGTTCACGGGGTC3′	18-mer primer which is complementary to nucleotides 2061 to 2078 in E. coli rRNA and could be extended to analyze the state of methylation at A2058 after annealing.
The upper strand sequence of the synthesized DNA fragment for 72 nt RNA substrate	5′G**GAATTC**taatacgactcactataGGCCTATCCGTCGCGGG***TTCG***CCCGCGACAGGACGGAAAGACCCCGTGGAGCTT***TTCG***AAGCTACCCCGGGGgccggccatggtcccagcctcctcgctggcggccggtgggcaacattccgaggggaccgtcccctcggtaatggcgaatgggac**TCTAGA**GC3′	The upper strand sequence of the synthesized DNA fragment alone (188 bp, bold letters denote *Eco*RI and *Xba*I recognition site, respectively. T7 promoter sequence in underlined lower case letter, UUCG tetraloop in bold, italicized uppercase and HDV ribozyme sequence in lower case.)

### Construction of expression vector.

The *ermK* gene was planned to be cloned into the NdeI and XhoI sites of pET23b for its overexpresssion. However, the NdeI site could be found in *ermK* structural gene and site directed mutagenesis of the *ermK* gene without any amino acid change (GC*A*TATGTG, which code 158AYV160 but as could be seen in underlined letters, it contains NdeI recognition site, so it changed to C*T*TATG as denoted in italicized letter.) was carried out using a sequential PCR method, known as the “splicing by overlap extension” (31, 32). In brief, to obtain the DNA fragment encompassing the 5′ end region, including the mutagenizing site, PCR was carried out using Bacillus halodurans C-125 chromosomal DNA as a template and the oligonucleotides oligo-1 and oligo-2 (Table 2) as the forward and reverse primers. The second PCR was performed using oligo-3 and oligo-4 as the forward and reverse primers, respectively, to obtain the 3′ end region of the *ermK* gene, including the mutagenizing site. When the two DNA fragments were obtained, which contained the overlapping region (italicized letters in Table 2) and the mutated nucleotide (bold letter in Table 2), they were mixed together and subjected to a third PCR in the presence of two primers, oligo-1 and oligo-4, to obtain the full-length *ermK* gene containing the desired mutation. The resultant PCR product was directly digested with NdeI and XhoI restriction enzymes and ligated into pET23b NdeI-XhoI sites. The cloned gene was sequenced to confirm the sequence and frame of the inset and named pHJJ602. The *ermC′* gene was also cloned from pIM13 (33) by PCR using oligo-5 and oligo-6 as the forward and reverse primers, respectively. The resultant fragment was treated as above to get expression vector (pHJJ502) for *ermC′*.

### Protein expression and purification.

Each Erm protein was expressed and purified from E. coli BL21(DE3) harboring plasmids pHJJ502 (ErmC′) and pHJJ602 (ErmK) as described previously (27, 34) with slight modifications. Briefly, transformed E. coli BL21(DE3) cells with the *ermC′* or *ermK* genes in the pET23b vector (E. coli HJJ502 and E. coli HJJ602) were grown overnight at 37°C in LB medium supplemented with 100 μg/mL of ampicillin. The cells were then transferred to fresh LB medium (10%, vol/vol) and incubated at 37°C for another 1.5 h to reach an A600 of 0.8 to 1.0. In order to induce the expression, IPTG (isopropyl-β-d-thiogalactopyranoside) was added to a final concentration of 1 mM and incubation was continued for another 18 h at 22°C. To analyze the leaky expression, incubation was carried out without IPTG induction. The resultant culture was centrifuged to collect the cells. After resuspension in buffer A (20 mM Tis-HCl [pH 7.5], 10 mM Mg Cl_2_, 500 mM KCl, and 5 mM imidazole) containing lysozyme (5 mg/mL), cells were incubated at room temperature for 20 min. After incubation, freezing and thawing was carried out to disrupt the cell. After treatment with DNase I and RNase A, the lysate was centrifuged at 20,000 × *g* for 30 min to separate the soluble proteins from the insoluble proteins (inclusion body fraction). To evaluate the relative quantity of solubilized and aggregated forms of ErmC′ and ErmK in E. coli cells, the separated fractions were analyzed by SDS-PAGE. The soluble protein fraction was loaded onto a column containing His.Bind resin preequilibrated with buffer A. The column was washed extensively with buffer B (20 mM Tris-HCl [pH 7.5], 10 mM MgCl_2_, 500 mM KCl, and 100 mM imidazole). The target protein was eluted with buffer C (20 mM Tris-HCl [pH 7.5], 10 mM MgCl_2_, 500 mM KCl, and 300 mM imidazole). To remove the imidazole, the eluted protein solution was passed through a PD-10 desalting column, as described by GE Healthcare (Little Chalfont, Buckinghamshire, UK) and then stored at −20°C in 20 mM Tris-HCl (pH 7.5), 10 mM MgCl_2_, 500 mM KCl, and 50% glycerol. The protein concentration was determined by the bicinchonic acid (BCA) protein assay method (Pierce, Rockford, IL, USA).

### Cloning for *in vitro* transcription of substrate RNA and *in vitro* transcription.

The DNA fragment encoding the 72 nt RNA substrate sequence (Fig. 5) was synthesized (Bionics, Seoul, South Korea) which is equipped with an EcoRI recognition sequence and a T7 promoter sequence at the 5′ end and the hepatitis delta virus (HDV) ribozyme sequence and XbaI recognition sequence at the 3′ end. The attached HDV ribozyme sequence functions to generate the homogeneous 3′ end of produced RNA by *in vitro* transcription (35). The obtained DNA fragment was cloned into the multicloning sites of EcoRI and XbaI in pUC19. The plasmid was linearized with XbaI for runoff transcription. The linearized plasmid was used directly as the template for the synthesis of substrate RNA transcripts with T7 RNA polymerase. Transcription from the linearized plasmid was performed as described previously (27). Briefly, transcription was carried out at 37°C for 4 h in the mixture containing 40 mM Tris-HCl (pH 8.1), 5 mM dithiothreitol (DTT), 1 mM spermine, 0.01% Triton X-100, 80 mg/mL PEG, 25 μg of DNA template, 4 mM ribonucleoside triphosphates (rNTPs), 28 mM MgCl_2_, and 10 μg of T7 RNA polymerase (prepared in-house). After transcription, the transcript was purified with phenol-chloroform, then ethanol precipitation and the self-cleavage reaction by the 3′ HDV ribozyme was performed (35). After the integrity was checked and the size was verified on 7 M urea-polyacrylamide gel, the correct band was extracted and self-folded to measure the methyl group accepting activity.

### *In vitro* methylation assay.

*In vitro* methylation of the 72 nt RNA substrate using the Erm protein was carried out using a slightly modified version of a previously described procedure (27). The reaction was performed in 50 μL volumes containing 50 mM Tris-HCl (pH 7.5), 4 mM MgCl_2_, 40 mM KCl, 10 mM dithiothreitol, 3.3 pmol SAM (specific activity, 80 Ci/mmol; PerkinElmer), 10 pmol 72 nt RNA substrate, and 10 pmol purified Erm proteins for 1 h. Reaction mixtures containing everything except proteins were prewarmed to 37°C by at least 5 min of incubation, then the purified Erm proteins were added to prewarmed tubes to minimize any lag in the start of the reaction. After 1 h of incubation, 0.5 mL of ice cold 12% trichloroacetic acid was added in order to terminate the reaction. The methylated RNAs collected by centrifugation were washed twice with 1.25 mL of ice-cold 6% trichloroacetic acid. After drying, the precipitate was extracted with 3 mL of scintillation fluid (Ultima Gold; Packard) and transferred to a counting vial. The remaining precipitate was extracted again with 75 μL of double-distilled water (DDW) warmed to 50°C to 60°C and then extracted once again with 25 μL of prewarmed DDW. All the extracts were pulled together, mixed well, and counted (Tri-Carb 2900TR; Packard, Shelton, CT, USA). Experiments were repeated at least thrice.

### *In vivo* activity assay of Erm proteins expressed in E. coli.

The activity of each Erm protein expressed in E. coli was evaluated as described in the previous report with slight modifications (32). Briefly, each E. coli culture (E. coli HJJ402, E. coli HJJ502, and E. coli HJJ602) was spread on quartered LB agar plate. Dried filter paper circles containing 1,000 μg erythromycin or tylosin were placed in the middle of the cultures. They were then incubated overnight at 37°C and the inhibition zone was observed, which formed by the inhibitory action of the antibiotics.

### Analysis of the degree of methylation through ErmK by reverse transcription.

Transformed E. coli cells (E. coli HJJ502, E. coli HJJ402, E. coli HJJ602 and E. coli BL21[DE3] harboring pET23b) were grown overnight at 37°C, then transferred to fresh LB medium (10%, vol/vol) and incubated at 37°C for 1 h to reach an A600 of 0.8 to 1. In order to induce overexpression, IPTG was added to a final concentration of 1 mM and incubation was continued further for another 6 h at 37°C. To analyze the degree of methylation with leaky expressed Erm proteins, E. coli cells were grown without IPTG induction. Subsequently, total RNA extraction using 3 mL of the culture obtained above was carried out by a single extraction with an acid guanidinium thiocyanate-phenol-chloroform mixture (36). The degree of methylation of RNA from each strain was analyzed by the primer extension method developed by Sigmund et al. (37). A 5′ end-labeled deoxyoligonucleotide primer (oligo-7, [38]) with ^32^P, which is complementary to nucleotides 2061 to 2078 in E. coli 23S rRNA (Fig. 4a, [39]) was extended with reverse transcriptase (BeamsBiotechnology, SungNam, South Korea) together with 1 mM dTTP and ddCTP for 20 min at 42°C. The extension products were run on a denaturing 13% polyacrylamide gels alongside dideoxy sequencing reactions performed on an unmodified rRNA template. Dimethylation at A2058 gave rise to a new band (Fig. 4b), the relative intensity of which was measured by scanning exposed autoradiographs with Multi Gauge V3.0 (Fujifilm Corporation, Tokyo, Japan).
