# Supplementary material for: Characteristics of the ErmK Protein of Bacillus halodurans C-125
Source: Microbiol Spectr. 2022 Dec 13;11(1):e02598-22. doi: 10.1128/spectrum.02598-22 (PMC9927578; doi:10.1128/spectrum.02598-22)
Supplement: Supplemental file 1 — Fig. S1. Download spectrum.02598-22-s0001.pdf, PDF file, 0.2 MB [file spectrum.02598-22-s0001.pdf]

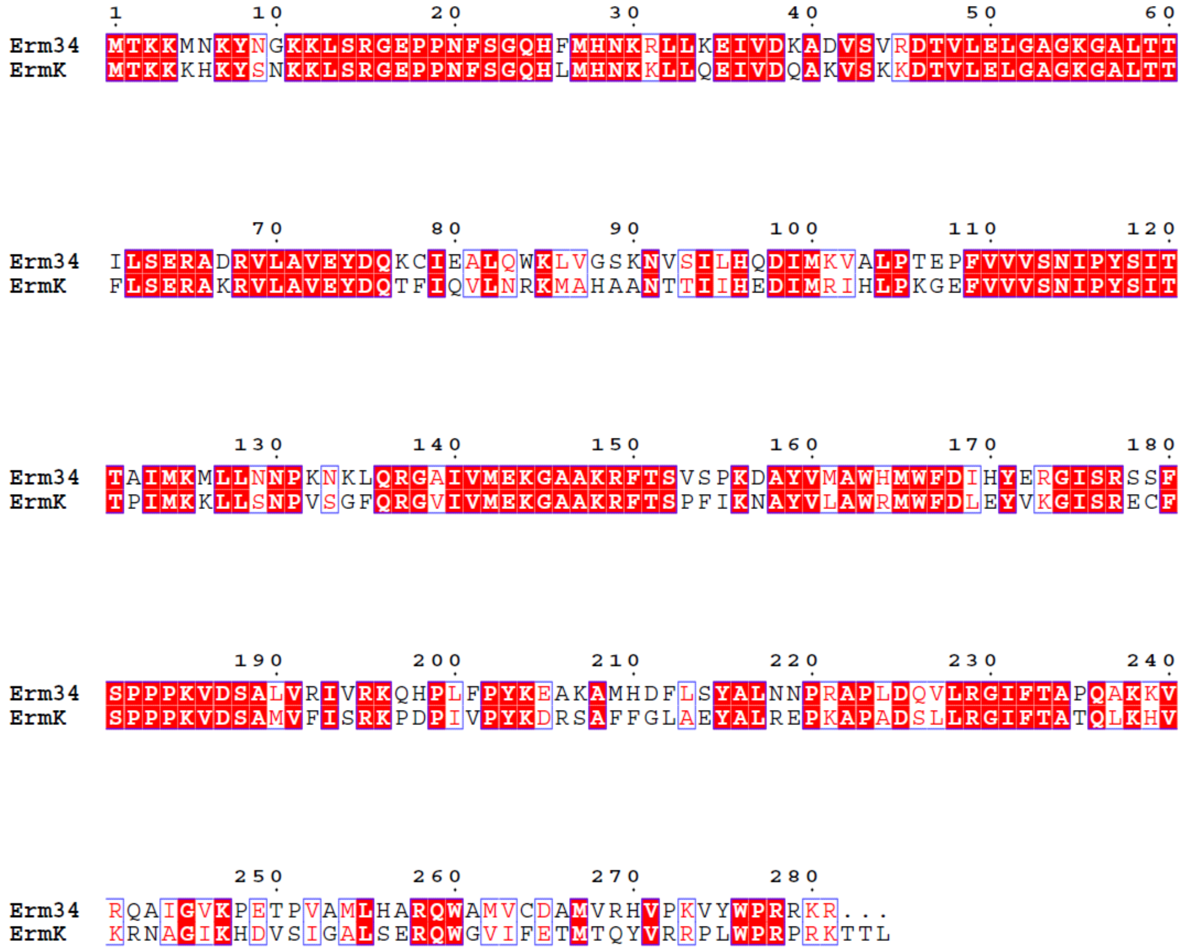

Figure S1. Sequence alignment of ErmK and Erm(34). Sequence alignment between ErmK and Erm(34) showed that two proteins have 61.2% amino acid sequence identity. So, ErmK could be classified as a new member of Erm protein and given a new letter designation of ErmK correctly.
